# Lower blood pressure and risk of cisplatin nephrotoxicity: a retrospective cohort study

**DOI:** 10.1186/s12885-017-3135-6

**Published:** 2017-02-20

**Authors:** Kazumi Komaki, Tetsuro Kusaba, Mai Tanaka, Hiroshi Kado, Yayoi Shiotsu, Masahiro Matsui, Atsushi Shiozaki, Hiroshi Nakano, Takeshi Ishikawa, Hitoshi Fujiwara, Hideyuki Konishi, Yoshito Itoh, Satoaki Matoba, Keiichi Tamagaki

**Affiliations:** 1 Department of Nephrology, Graduate School of Medical Science, Kyoto Prefectural University of Medicine, 465 Kajii-cho, Kamigyo-ku, Kyoto, 602-8566 Japan; 2 Department of Otolaryngology-Head and Neck Surgery, Kyoto Prefectural University of Medicine, 465 Kajii-cho, Kamigyo-ku, Kyoto, 602-8566 Japan; 3 Division of Digestive Surgery, Department of Surgery, Kyoto Prefectural University of Medicine, 465 Kajii-cho, Kamigyo-ku, Kyoto, 602-8566 Japan; 4 Molecular Gastroenterology and Hepatology, Graduate School of Medical Science, Kyoto Prefectural University of Medicine, 465 Kajii-cho, Kamigyo-ku, Kyoto, 602-8566 Japan; 5 Department of Cardiovascular Medicine, Graduate School of Medical Science, Kyoto Prefectural University of Medicine, 465 Kajii-cho, Kamigyo-ku, Kyoto, 602-8566 Japan

**Keywords:** Blood pressure, Cisplatin, Food intake, Nephrotoxicity, Renin-angiotensin system (RAS) inhibitor

## Abstract

**Background:**

The pathophysiological mechanisms of cisplatin nephrotoxicity include the reduction of renal blood flow, as well as tubular epithelial cell toxicity. The objective of this study was to investigate the influence of lower blood pressure and decreased food intake on the incidence of cisplatin nephrotoxicity.

**Methods:**

We conducted a retrospective cohort study at a university hospital between 2011 and 2012. We identified hospitalized adult patients with head and neck cancer, esophageal cancer, or gastric cancer, who received intravenous cisplatin administration. The primary outcome was the incidence of cisplatin nephrotoxicity defined as the increase in serum creatinine after cisplatin administration more than 1.5 times from baseline.

**Results:**

The study participants included 182 patients, in whom we observed a total of 442 cycles of cisplatin chemotherapy. The incidence of cisplatin nephrotoxicity was observed in 41 of 182 cycles with initial administration. Multivariate logistic regression analysis showed that systolic blood pressure was independently associated with cisplatin nephrotoxicity (adjusted odds ratio 0.75, 95% confidence interval 0.57 to 0.95 for each 10 mmHg). The use of renin-angiotensin system (RAS) inhibitors was also associated with cisplatin nephrotoxicity (3.39, 1.30 to 8.93). Among quartiles of systolic blood pressure in all cycles of chemotherapy, the incidence of nephrotoxicity in the lower blood pressure group was significantly higher than that in the higher blood pressure group for patients taking non-solid food (*P* = 0.037), while there was no significant difference for patients taking solid food (*P* = 0.67).

**Conclusions:**

Lower blood pressure and the use of RAS inhibitors were associated with the incidence of cisplatin nephrotoxicity, and lower blood pressure had a greater influence on nephrotoxicity in patients who could not take solid food. Discontinuation of antihypertensive medication including RAS inhibitors before cisplatin chemotherapy should be considered, which may be beneficial for patients with lower blood pressure.

**Electronic supplementary material:**

The online version of this article (doi:10.1186/s12885-017-3135-6) contains supplementary material, which is available to authorized users.

## Background

Cisplatin is a platinum-based anticancer drug widely used to treat various types of cancer and contributes to the improvement in outcomes like 5-year survival. However, side effects of cisplatin including ototoxicity, neurotoxicity, nausea, and myelosuppression frequently occur, and the main dose-limiting side effect is nephrotoxicity [1, 2]. Previous reports showed that cisplatin nephrotoxicity occurred in approximately 7–29% of patients and several risk factors were reported [3–6]. For example, de Jongh et al. reported that age, female gender, smoking, paclitaxel co-administration, hypoalbuminemia were the risk factors of cisplatin nephrotoxicity in 400 patients with advanced solid tumors [5]. It was also reported that higher plasma platinum concentrations, hyperuricemia, and hypoalbuminemia were associated with renal dysfunction due to cisplatin [7–9].

Though hydration, monitoring of renal function, and adjustment of cisplatin doses depending on renal function [10, 11] are commonly performed in usual clinical practice, specific therapeutic approaches for the prevention and treatment of cisplatin nephrotoxicity has not been established yet. To ameliorate cisplatin nephrotoxicity, numerous approaches such as blocking inflammation, injury signaling, and cell death pathway have been reported in animal models or cultured cells [12–14]. However, whether these approaches are applicable to human patients is still unknown [1, 10].

The pathophysiological mechanisms of cisplatin nephrotoxicity include its direct tubular epithelial cell toxicity as well as the reduction of renal blood flow as a consequence of endothelial dysfunction and vasoconstriction [15, 16]. However, there are few reports in literature that have assessed the influence of hemodynamic conditions such as lower blood pressure and decreased food intake on cisplatin nephrotoxicity [17]. Thus, we hypothesized that risk factors such as lower blood pressure and decreased food intake deteriorate cisplatin nephrotoxicity by reducing renal blood flow despite routine administration of hydration.

In this study, we retrospectively reviewed a cohort of patients treated with cisplatin-based chemotherapy for head and neck cancer, esophageal cancer, or gastric cancer. The subject selection criteria were determined according to the following two reasons: patients with these types of cancer tend to decrease their food intake from trismus, dysphagia, or gastrointestinal symptoms and these cancers are similar in cisplatin dosage and administration interval. The objective of this study was to investigate the influence of lower blood pressure and decreased food intake on the incidence of cisplatin nephrotoxicity.

## Methods

### Study design and participants

We conducted a retrospective cohort study at University Hospital, Kyoto Prefectural University of Medicine, Kyoto, Japan. By searching electronic medical records, we identified hospitalized patients aged 18 years or older with head and neck cancer, esophageal cancer, or gastric cancer, who received intravenous cisplatin administration between January 2011 and December 2012. The following patients were excluded from the study: patients who received cisplatin before the observation period, those with a history of previous cisplatin administration at other hospitals, those with an interval of cisplatin administration of less than 2 weeks, and those receiving maintenance dialysis.

We analyzed the incidence of cisplatin nephrotoxicity in the first cycle of cisplatin chemotherapy and the relationship of potential risk factors to the incidence of cisplatin nephrotoxicity. Then, we evaluated all cycles of cisplatin chemotherapy during the observation period to investigate the relationship of lower blood pressure and decreased food intake to the incidence of cisplatin nephrotoxicity, because these factors can vary among cycles of chemotherapy even in the same patient. The study was approved by the Ethics Committee on Human Research of Kyoto Prefectural University of Medicine and was carried out in accordance with the Declaration of Helsinki. Patient records/information was anonymized and de-identified prior to analysis.

### Outcomes and follow-up

The primary outcome was the incidence of cisplatin nephrotoxicity defined as the increase in serum creatinine after cisplatin administration more than 1.5 times baseline according to the Common Terminology Criteria for Adverse Events (CTCAE) version 4.0 [18]. We used serum creatinine, measured prior to each cycle of cisplatin administration as the baseline value, and collected the highest serum creatinine in the first 4 weeks of the cycle.

### Data collection

Baseline characteristics of patients were extracted from electronic medical records as follows: age, sex, smoking status (current and former, never), history of hypertension, history of diabetes, history of cardiovascular disease, cancer types, and combined anticancer drugs. Cardiovascular disease was defined as angina or myocardial infarction, referring to a previous report [19]. The clinical parameters below were collected at each cycle of chemotherapy: height, weight, body mass index (BMI), body surface area (BSA), cisplatin dose, cumulative cisplatin dose, cycle number, combination of anticancer drugs, amount of hydration, diuretics, food form (solid, non-solid), amount of food intake, and antihypertensive medications including calcium channel blockers and renin-angiotensin system (RAS) inhibitors. We served food containing 1600–1800 kcal, 6–9 g of NaCl daily and less than 30% of food intake was arbitrarily defined as “low food intake”. Non-solid food was defined as liquid food or food which was minced or pasted. Non-solid food was served when the patients could not eat solid food because of nausea or gastro-intestinal obstruction due to the cancer. All patients received the drug within the manufacturer’s recommended dose and the hydration protocol that is routine in our institute. We collected systolic and diastolic blood pressure and baseline laboratory data such as serum creatinine, C-reactive protein (CRP), serum albumin, and hemoglobin measured prior to cisplatin administration.

### Statistical analysis

Data are shown as number (percentage) for categorical variables and mean ± standard deviation (SD) for continuous variables. Categorical variables were compared using chi-square tests or Fisher’s exact tests for small sample sizes. Chi-square test with Bonferroni correction was used for multiple comparisons. Continuous variables were compared using a Welch’s *t* test.

Multivariate logistic regression analysis was performed to evaluate the influence of clinical variables on cisplatin nephrotoxicity in the first cycle of chemotherapy. The variables included age, sex, cisplatin dose per BSA, food form (solid, non-solid), systolic blood pressure, and the use of RAS inhibitors. Data of logistic regression analysis are given as adjusted odds ratio (OR) with a 95% confidence interval (CI) and *P* value.

To examine the relationship between blood pressure and nephrotoxicity, all cycles were grouped into quartiles based on systolic blood pressure. The incidence of nephrotoxicity and the prevalence of antihypertensive medication use in each group were calculated. Then, the relationship between cisplatin nephrotoxicity, quartiles of systolic blood pressure, and food form (solid or non-solid food) were analyzed.

Differences were determined to be significant when the two-sided *P* value was less than 0.05. Statistical analyses were performed using JMP software, Version 10 (SAS Institute Inc., Cary, NC).

## Results

### Study participants

**Fig. 1:**
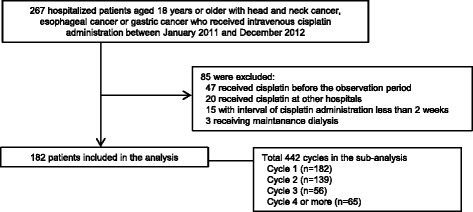
Flowchart of participants analyzed in this study

During the study period, 267 patients were assessed for eligibility. Figure 1 shows the flowchart of study participants. We excluded 85 patients from analysis due to following reasons: 47 received cisplatin before the observation period, 20 received cisplatin at other hospitals, 15 with interval of cisplatin administration less than 2 weeks, and 3 receiving maintenance dialysis. As a result, the study participants included 182 patients (135 men, 47 women), in whom we observed a total of 442 cycles of cisplatin administration (182 cycle 1, 139 cycle 2, 56 cycle 3, 65 cycle 4 or more). The baseline characteristics for study participants are listed in Table 1; mean age was 65.1 years, and 74.2% were men. Cancer types were head and neck cancer (42.3%), esophageal cancer (45.1%), and gastric cancer (12.6%).

**Table 1:** Baseline characteristics for study participants

Characteristic	All patients (*n* = 182)
Age (years)	65.1 ± 9.4
Male sex	135 (74.2)
Smoker	139 (76.4)
Hypertension	67 (36.8)
Antihypertensive medication	51 (28.0)
Calcium channel blockers	35 (19.2)
RAS inhibitors	31 (17.0)
Others	14 (7.7)
Diabetes	25 (13.7)
Cardiovascular disease	10 (5.5)
Cancer types	
Esophagus	82 (45.1)
Head and neck	77 (42.3)
Stomach	23 (12.6)
Combined anticancer drugs	138 (75.8)
5-FU	108 (59.3)
TS-1	19 (10.4)
DOC	14 (7.7)
CPT-11	5 (2.7)
Capecitabine	5 (2.7)

Data are shown as number (percentage) or mean ± standard deviation. *RAS* renin-angiotensin system, *5-FU* 5-fluorouracil, *TS-1* tegafur gimeracil oteracil potassium, *DOC* docetaxel, *CPT-11* irinotecan

### Development of cisplatin nephrotoxicity

The incidence of cisplatin nephrotoxicity was observed in 41 of 182 cycles with initial administration, in which 14 patients discontinued following cisplatin chemotherapy. In addition, cisplatin nephrotoxicity was observed in 71 of the total 442 cycles; 8 patients developed multiple episodes of nephrotoxicity (once: 54 patients, twice: 7 patients, three times: 1 patient).

### Risk factors for cisplatin nephrotoxicity

To investigate the relevant factors for developing cisplatin nephrotoxicity, we compared the clinical characteristics for patients with and without subsequent nephrotoxicity in the first cycle (Table 2). Systolic blood pressure was significantly lower and the use of RAS inhibitors was significantly higher in the group with subsequent nephrotoxicity. There was no statistically significant difference in cisplatin dose, amount of hydration, non-solid food, or decreased food intake between the two groups.

**Table 2:** Clinical characteristics for patients with and without subsequent nephrotoxicity in the first cycle

Characteristic	Nephrotoxicity (+)	Nephrotoxicity (−)	*P* value
	(*n* = 41)	(*n* = 141)	
Age (years)	65.7 ± 9.0	65.0 ± 9.6	0.69
Male sex	33 (80.5)	102 (72.3)	0.12
BMI (kg/m^2^)	20.4 ± 3.2	21.0 ± 3.3	0.30
Systolic blood pressure (mmHg)	114.3 ± 15.7	119.8 ± 15.4	0.0498
Diastolic blood pressure (mmHg)	68.6 ± 9.1	71.7 ± 11.4	0.08
Antihypertensive medication	14 (34.2)	37 (26.2)	0.32
Calcium channel blockers	9 (22.0)	26 (18.4)	0.62
RAS inhibitors	12 (29.3)	19 (13.5)	0.02
Others	5 (12.2)	9 (6.4)	0.22
Cardiovascular disease	1 (2.4)	9 (6.4)	0.33
Combination of anticancer drugs	32 (78.1)	106 (75.2)	0.71
Cisplatin dose (mg/m^2^)	73.4 ± 9.6	69.8 ± 13.7	0.054
Amount of hydration (mL/day)	3,437 ± 319	3,345 ± 595	0.20
Diuretics	38 (92.7)	127 (90.1)	0.61
Non-solid food	21 (51.2)	50 (35.5)	0.07
Decreased food intake (≤50%)	9 (22.0)	18 (12.8)	0.15
Laboratory data			
Creatinine (mg/dL)	0.69 ± 0.19	0.70 ± 0.17	0.83
CRP (mg/dL)	1.01 ± 1.77	0.87 ± 1.77	0.66
Albumin (g/dL)	3.80 ± 0.51	3.91 ± 0.49	0.22
Hemoglobin (g/dL)	12.7 ± 1.6	12.7 ± 1.8	0.83

Data are shown as number (percentage) or mean ± standard deviation. *BMI* body mass index, *RAS* renin-angiotensin system, *CRP* C-reactive protein

To further investigate the risk factors for developing nephrotoxicity, we performed multivariate logistic regression analysis and found that systolic blood pressure was independently associated with cisplatin nephrotoxicity (adjusted OR 0.75, 95% CI 0.57 to 0.95 for each 10 mmHg, *P* = 0.020; Table 3). In addition, the use of RAS inhibitors was observed in 17.0% of the cycles and was associated with cisplatin nephrotoxicity (3.39, 1.30 to 8.93, *P* = 0.013).

**Table 3:** Multivariate logistic regression analysis of clinical variables for cisplatin nephrotoxicity in the first cycle

Variable	Adjusted OR (95%CI)	*P* value
Age	1.00 (0.96–1.05)	0.87
Male sex	1.91 (0.79–5.05)	0.15
Cisplatin dose, 10 mg/m^2^	1.29 (0.92–1.87)	0.14
Non-solid food	2.09 (0.99–4.50)	0.054
Systolic blood pressure, 10 mmHg	0.75 (0.57–0.95)	0.02
RAS inhibitors use	3.39 (1.30–8.93)	0.01

Multivariate logistic regression analysis was performed to evaluate the influence of clinical variables on cisplatin nephrotoxicity in the first cycle of chemotherapy (*n* = 182)

*OR* odds ratio, *CI* confidence interval, *RAS* renin-angiotensin system

### Lower blood pressure as a risk of cisplatin nephrotoxicity

Next, we closely focused on the relationship between blood pressure and decreased food intake to the incidence of cisplatin nephrotoxicity. We evaluated all cycles of chemotherapy during the observation period, because the hemodynamic conditions can vary among cycles of chemotherapy even in the same patient. Systolic blood pressure was significantly lower and the proportion of patients who were taking non-solid food was significantly higher in the group with subsequent nephrotoxicity (Table 4).

**Table 4:** Clinical characteristics for all cycles of chemotherapy with and without subsequent nephrotoxicity

Characteristic	Nephrotoxicity (+)	Nephrotoxicity (−)	*P* value
	(*n* = 71)	(*n* = 371)	
BMI (kg/m^2^)	20.0 ± 3.3	20.6 ± 3.1	0.12
Systolic blood pressure (mmHg)	112.0 ± 14.7	118.5 ± 15.1	<0.001
Diastolic blood pressure (mmHg)	68.3 ± 9.7	71.6 ± 11.1	0.01
Antihypertensive medication	20 (28.2)	80 (21.6)	0.22
Calcium channel blockers	13 (18.3)	58 (15.7)	0.60
RAS inhibitors	14 (19.7)	43 (11.6)	0.08
Others	7 (9.9)	18 (4.9)	0.10
Combination of anticancer drugs	54 (76.1)	299 (80.6)	0.42
Cisplatin dose (mg/m^2^)	71.4 ± 10.5	68.2 ± 13.3	0.03
Cumulative cisplatin dose (mg/m^2^)	135 ± 83	152 ± 101	0.13
Amount of hydration (mL/day)	3,441 ± 343	3,391 ± 532	0.31
Diuretics	66 (93.0)	345 (93.2)	0.93
Non-solid food	38 (53.5)	141 (38.0)	0.02
Decreased food intake (≤50%)	12 (16.9)	62 (16.7)	0.97
Laboratory data			
Creatinine (mg/dL)	0.70 ± 0.20	0.74 ± 0.21	0.12
CRP (mg/dL)	1.04 ± 1.65	0.66 ± 1.45	0.08
Albumin (g/dL)	3.83 ± 0.46	3.87 ± 0.45	0.43
Hemoglobin (g/dL)	12.1 ± 1.6	11.9 ± 1.8	0.37

Data are shown as number (percentage) or mean ± standard deviation. *BMI* body mass index, *RAS* renin-angiotensin system, *CRP* C-reactive protein

**Fig. 2:**
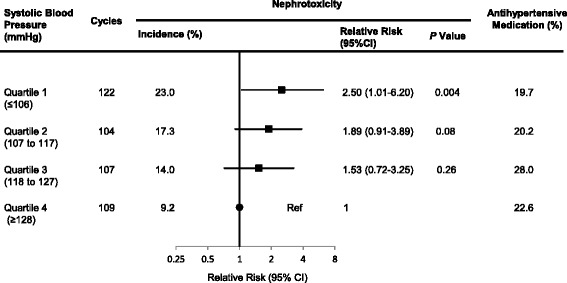
Incidence of nephrotoxicity and prevalence of antihypertensive medication use by quartiles of systolic blood pressure. All cycles were divided into quartiles of systolic blood pressure (*n* = 442). CI, confidence interval

Then, we divided all cycles into quartiles of systolic blood pressure. As demonstrated in Fig. 2, the incidence of nephrotoxicity was significantly higher in the lower quartile than in the higher quartile (relative risk 2.50, 95% CI 1.01 to 6.20, *P* = 0.004). Despite low blood pressure, approximately 20% of subjects continued antihypertensive medication in the lower quartile. Additionally, there was no significant difference in the prevalence of antihypertensive medication use between quartiles.

**Fig. 3:**
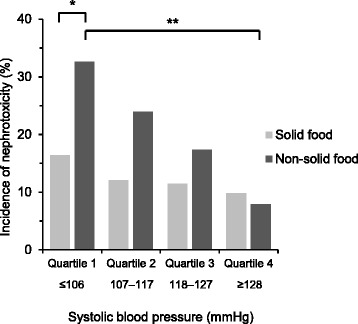
Incidence of nephrotoxicity according to food form and quartiles of systolic blood pressure. All cycles were classified according to food form (solid or non-solid food) and quartiles of systolic blood pressure (*n* = 442). Chi-square test revealed as; *significant differences between patients taking solid food and non-solid food in Quartile 1 (*P* < 0.05), and **significant differences between Quartile 1 and Quartile 4 in patients taking non-solid food (*P* < 0.0125 after Bonferroni correction)

Finally, we classified all cycles according to food form (solid or non-solid food) and quartiles of systolic blood pressure (Quartile 1: ≤106 mmHg, Quartile 2: 107 to 117 mmHg, Quartile 3: 118 to 127 mmHg, Quartile 4: ≥128 mmHg), and compared the incidence of cisplatin nephrotoxicity among these groups (Fig. 3). For patients taking solid food, the incidence of cisplatin nephrotoxicity in the lower blood pressure group was slightly higher than that in the higher blood pressure group (Quartile 1 = 16.4%, Quartile 2 = 12.1%, Quartile 3 = 11.5%, Quartile 4 = 9.9%, *P* = 0.67). By contrast, for patients taking non-solid food, the incidence of nephrotoxicity in the lower blood pressure group was significantly higher than that in the higher blood pressure group (Quartile 1 = 32.7%, Quartile 2 = 23.9%, Quartile 3 = 17.4%, Quartile 4 = 7.9%, *P* = 0.037). Lower blood pressure had a greater influence on nephrotoxicity in patients taking non-solid food than those taking solid food. For the lower quartile (Quartile 1), the incidence of nephrotoxicity in patients taking non-solid food was significantly higher than that in patients taking solid food (relative risk 1.98, 95% CI 1.03 to 3.82, *P* = 0.037).

## Discussion

This retrospective cohort study addresses the risk of cisplatin nephrotoxicity and especially focuses on the impact of lower blood pressure and decreased food intake. In this study, we verified that lower blood pressure prior to cisplatin administration, and the use of RAS inhibitors were associated with the incidence of cisplatin nephrotoxicity. We also showed that non-solid food intake is a risk of cisplatin nephrotoxicity in case of coincidence with lower blood pressure.

Our study showed that lower blood pressure, especially lower than 107 mmHg, prior to cisplatin administration was a significant risk for the incidence of cisplatin nephrotoxicity. In terms of cisplatin metabolism, the unbound cisplatin in the plasma is freely filtered by the glomerulus and is not reabsorbed [11, 20]. On the other hand, circulating cisplatin is transported into proximal tubular epithelial cells by the organic cation transporter 2 (OCT2) which is highly expressed in the basolateral membrane of proximal tubules, which depends on the serum cisplatin concentration [21]. The renal accumulation of cisplatin causes direct tubular epithelial cytotoxicity [22, 23]. In patients with lower blood pressure, it is expected that urinary excretion of cisplatin is delayed by a decreased glomerular filtration rate (GFR). As a result, it takes a longer time for the serum cisplatin concentration to drop off and for cisplatin uptake into tubular epithelial cells through OCT2 to increase, which eventually accelerates cisplatin nephrotoxicity. The pathophysiology of cisplatin nephrotoxicity also involves vascular injury such as microangiopathy and vasoconstriction, which causes a reduction of renal blood flow and GFR [16, 17]. Moreover, it is reported that an increase of sodium excretion in urine and polyuria occur after cisplatin administration [24, 25], which induce further reduction of blood volume and a subsequent decrease in renal blood flow.

Regarding antihypertensive drugs, this study also suggests that the use of RAS inhibitors was associated with cisplatin nephrotoxicity independent of systolic blood pressure. RAS is activated when the renal perfusion pressure is decreased by hypotension or volume depletion [26]. Angiotensin II increases systemic blood pressure by contracting arterioles, promotes sodium reabsorption in renal tubules, and increases extracellular fluid [27]. Consequently, angiotensin II maintains glomerular filtration pressure and GFR [26]. An experimental study using dogs showed a dissociation of autoregulation in renal blood flow and GFR was introduced by RAS inhibitors; GFR dramatically dropped off according to the decrease in renal perfusion pressure while renal blood flow was preserved in the same condition [28–30]. In fact, patients taking RAS inhibitors are at high risk of developing acute kidney injury during intercurrent illnesses such as volume depletion, because the contraction of efferent arterioles is inhibited by RAS inhibitors [31]. In addition, RAS can be activated by volume depletion from sodium wasting or polyuria and vascular contraction after cisplatin administration. It was reported that plasma renin activity and plasma aldosterone concentrations were elevated after cisplatin administration [32]. RAS inhibitors may suppress this humoral response, resulting in exacerbating renal ischemia and delaying cisplatin excretion by inhibiting RAS activation to maintain GFR.

On the other hand, Saleh et al. reported that the angiotensin receptor blocker losartan has protective effects against cisplatin-induced nephrotoxicity in a rat model [33]. In that study, losartan did not affect cisplatin uptake by the kidney, but significantly counteracted cisplatin-induced lipid peroxidation and glutathione depletion. They concluded that the renoprotective effect is due to antioxidant properties [33]. In our study, in subjects who tend to present with volume depletion from decreased food intake, the harmful effect on glomerular filtration pressure might exceed the antioxidative effect of RAS inhibitors. Further investigation is needed to address this issue.

Concerning food intake, the prevalence of appetite loss is high among cancer patients and various causes are involved. For example, emotional distress by the diagnosis or treatment [34], active inflammatory reaction caused by cancer, chemotherapy or irradiation may decrease the appetite [35]. Of note, patients of head and neck cancer frequently developed taste and smell disturbance because of cancer involvement in taste nerves or local taste bud injury caused by irradiation or surgery, which remarkably decrease the patient’s quality of life. Unless essential minerals are appropriately supplied, lower food intake reflects the reduction of sodium intake, resulting in a notable decrease in blood pressure and subsequent increase in the risk of cisplatin nephrotoxicity. In fact, our study demonstrated that non-solid food intake was a risk of cisplatin nephrotoxicity only in case of lower blood pressure. From these findings, we have to pay much attention to food intake and subsequent lower blood pressure in order to avoid cisplatin nephrotoxicity.

This study showed no significant association between cisplatin dose and nephrotoxicity in multivariate analysis. Reece et al. reported that the peak plasma level of ultrafilterable platinum correlated with a decline in creatinine clearance after cisplatin therapy in 22 cancer patients who received cisplatin [7]. Lagrange et al. also reported that platinum concentration was only related to the incident rate of nephrotoxicity and that platinum concentration was related to pretreatment renal function, BSA, cisplatin dose, number of administration in 121 cycles of 62 cancer patients who received cisplatin every 3 weeks [8]. In our institute, cisplatin dose is empirically reduced in the presence of pre-existing renal dysfunction [36]. It is suggested that cisplatin dose adjustment for renal impairment hides possible associations between cisplatin dose and nephrotoxicity in this study.

Our study has several limitations. First, our analysis is lacking an appropriate control group such as patients who maintained adequate food intake or those with higher blood pressure. Therefore, we classified patients according to food form and blood pressure, and evaluated their association with the outcome within a cohort. Second, the study also has the inherent limitations of a retrospective study. To quantify feeding status, we used two parameters: food form (solid, non-solid) and amount of food intake. The medical records of these data were partially semi-quantitative. The quantitative indicators of food intake or other parameters for nutritional status are needed for further investigation. Third, serum creatinine, used for primary outcome in this study, can overestimate basal renal function and underestimate renal prognosis in patients with weight loss due to cancer. To avoid the latter problem, we evaluated short-term creatinine changes within 4 weeks. Finally, there was the lack of longitudinal data including blood pressure and body weight. These data can elucidate the change in distribution of blood pressure and relevant factors in cancer patients.

## Conclusions

In conclusion, our study demonstrated that lower blood pressure prior to cisplatin administration and the use of RAS inhibitors was associated with the incidence of cisplatin nephrotoxicity, and lower blood pressure had a greater influence on nephrotoxicity in patients who could not take solid food because of nausea or gastro-intestinal obstruction due to the cancer. We wish to alert oncologists to the risk of cisplatin nephrotoxicity in cases of coincidence of lower blood pressure and decreased food intake. Discontinuation of antihypertensive medication including RAS inhibitors before cisplatin chemotherapy should be considered, which may be beneficial for patients with lower blood pressure.

## Additional file

Additional file 1: Original data of 442 cycles of cisplatin chemotherapy in 182 patients. (XLSX 88 kb)

## Abbreviations

5-FU: 5-fluorouracil
BMI: Body mass index
BSA: Body surface area
CI: Confidence interval
CPT-11: Irinotecan
CRP: C-reactive protein
CTCAE: Common Terminology Criteria for Adverse Events
DOC: Docetaxel
GFR: Glomerular filtration rate
OCT2: Organic cation transporter 2
OR: Odds ratio
RAS: Renin-angiotensin system
SD: Standard deviation
TS-1: Tegafur gimeracil oteracil potassium
